# Progress in Surgery

**Published:** 1898-11-19

**Authors:** 


### Progress in Surgery.

### SURGERY OF THE INTESTINES.

**Intussusception.**—S. W. Kelly[1] gives some practical points on the use of hydrostatic and pneumatic pressure in the reduction of intussusception. He refers to Forest's rule that eight or nine pounds' pressure is borne by the infant's intestine (an elevation of two and a half feet corresponding to one pound pressure to the square inch), and states his belief that a pressure of three to five pounds for twenty-five or thirty minutes is more efficient and safer than a pressure of seven or nine pounds for five or ten minutes. As regards the use of air, he considers that it has advantages, in that with air you feel more readily the giving way of the tumour, and further, that air will penetrate where water will not, viz., above the ileo-cæcal valve. Its disadvantage is that there is no way of practically measuring the pressure of air. Kelly obviates this difficulty by using air first and then introducing water after the air. The water pressure is readily measured by the elevation of the fountain, and, this following the air, it is to be presumed that the air pressure is equal to the water pressure. Saline solutions (a drachm to a quart) at a temperature of about 105 deg. are preferred, as being less irritating, tending to overcome shock, and being additionally useful should electricity be employed—one pole being applied to the saline solution in the rectum, and the other outside the abdomen over the tumour. Heaton[2] does not think that injections are harmless, for a fair number of cases has been reported in which rupture of the bowels has followed injection, and it is generally admitted that neither injection nor inflation should be employed after forty-eight or even twenty-four hours from the commencement of the illness, because of the formation of adhesions. He thinks that a primary laparotomy—that is, one not preceded by inflation or injection—offers the best chance of success, and quotes two cases of acute intussusception treated successfully by immediate abdominal section and reduction. Langton[3] records a case of ileo-cæcal intussusception cured by the excision of the involved intestine.

**Intestinal Obstruction**, according to Thomson,[4] varies in the importance of its symptoms. Vomiting is es-especially valuable, but the quality of the vomit must be considered, and the length of time it has continued. Persistent stercoraceous vomiting is an almost positive indication for operation. Pain is of value in locating the trouble within the abdomen, but it rarely indicates the actual site of obstruction. Temperature is often misleading, and must not therefore be relied upon. Constipation depends very much upon the position of the stoppage. If it is high up motions may persist for some time, and even diarrhœa may appear for a day or two. If the stoppage is in the large intestine we have the constipation from the outset, although sometimes a loaded rectum below the point may puzzle us by discharging its contents. Dulness in the flanks is a very unreliable sign. The presence of indican in the urine, and the suppression in proportion to the nearness of the stoppage to the beginning of the small intestine, are localising symptoms that are of value. In the vast majority of cases of acute obstruction, there is no room for medical treatment. Jones[5] records two cases of intestinal obstruction relieved by operation, and Thompson[6] give cases illustrative of obstruction produced by strangulation under Meckel's diverticulum.

**Intestinal Anastomosis** by means of rubber cylinders is advocated by Halsted.[7] The cylinder is introduced in a deflated condition, then inflated before suturing is commenced, and retained during the anastomosis until the process is almost completed. The bag is withdrawn, after being deflated, just before the insertion of the final sutures. The following advantages are claimed for its use in circular suture of the intestine: (1) The vermicular action of the intestine is arrested over the bag, and the stitches can consequently be placed at regular and proper intervals. (2) The distended bag unrolls and spreads out to a fine edge the everted raw edge of the intestine, and enables the operator to place the stitches with great precision at the desired distance from the edge. (3) If distended intestine is to be sutured to collapsed intestine, or intestine of larger to intestine of smaller lumen, the smaller may easily be expanded to fit the larger piece. (4) Very little handling of the intestine itself by the operator is necessary; the tube from bag to syringe is used as a handle to rotate and elevate the parts to be united. (5) The cylinder takes the place of at least two assistants, and the operation could readily be performed without an assistant. (6) It prevents escape of intestinal contents, and hence dispenses with injurious clamps or the fingers of assistants. (7) The entire operation, exclusive of suture of the abdominal wall, can be performed on dogs in five or six minutes, and probably in less time. Lockyer,[8] in describing a case of primary resection for intestinal gangrene, thinks that bone bobbins will ultimately prove to be of greatest service in aiding intestinal anastomosis. The objections to Murphy's button are: (1) Stenosis of the gut as a sequel (2) obstruction of the lumen; (3) failure to pass; (4) gangrene, extending from the seat of pressure, or set up by the button in its passage down the canal; (5) injury to the bowel during closure by the pressure of the fingers, causing the sharply-cut apertures for drainage to lacerate the mucous membrane; (6) impossibility of disarticulating the button once it is closed; (7) erosion of the spring by gastric juices. The chief merit of the button rests on the results.

**Tumours of the Large Intestine**, according to Vautrim,[9] are best treated by resection without fixation of the intestine to the abdominal parietes. The resection of the intestine should be accomplished by the thermocautery, whilst the mesentery should be cut with scissors, the arteries being caught up with forceps as soon as they are cut. In the performance of the enterorraphy sutures are rapid and certain, and are preferable to plates or buttons. The formation of an artificial anus should be reserved for cases of extreme urgency, where acute symptoms demand immediate interference. The resection should be performed secondarily, after the acute symptoms have subsided. When the tumour is inoperable, entero-anastomosis should be performed, wherever it is possible, with exclusion of the involved area. The author would not recommend the employment of the Murphy button in these operations, as the lumen of the button is too small compared with the calibre of the intestine, and he has seen it occluded. Resection is most commonly employed in tumours of the ascending and descending colon, while entero-anastomosis, with exclusion of the affected parts, is more common in the transverse colon. In tumours of small volume the loop of bowel containing the tumour may be drawn outside the abdomen, the intestine fixed in the wound, and the tumour resected later; this is followed by a later operation for the cure of the artificial anus. Knaggs[10] reports a case in which the cæcum and the ascending and transverse colon were successfully removed for malignant disease. The specimen measured ten and a half inches in length, and eleven inches in circumference at the thickest part.

**Idiopathic Dilatation of the Colon** is illustrated by Treves[11] by a case in which the entire rectum, sigmoid flexure, and descending colon were excised. This operation was performed on a child six years of age, who had suffered from constipation from birth. There was an enormous distension of the descending colon, the rectum and sigmoid flexure being only about the size of an adult's forefinger. An artificial anus was formed, but the child's condition was so distressing that nine months after the diseased parts were removed. The descending colon, sigmoid flexure, rectum, and anus were cut out, and the divided end of the transverse colon was brought down to and fixed in the position of the anus. The patient made a good recovery.

**Lumbar Colotomy**, as opposed to inguinal colotomy, is strongly advocated by Lawson Tait.[12] He says that he has never seen a case of lumbar colotomy where the beneficial effects were destroyed by the progress of the disease. An uncomfortable eversion of the gut after inguinal colotomy has been far more frequent and extensive than it ever is after the lumbar operation. Following McKenzie Edwards, he prefers a vertical incision about three inches long just outside the erector spinæ.

[1] New York Med. Jour., April 30, 1898, p. 611. [2] Lancet, June 4, 1898, p. 1537. [3] Brit. Med. Jour., May 21, 1898, p. 1327. [4] Amer. Jour. Med. Sci., April, 1898, p. 473. [5] Med. Chron. Mar., 1898, p. 408. [6] Annals of Surg., April, 1898, p. 448. [7] Brit. Med. Jour., April 2, 1898. [8] Lancet, April 30, 1898, p. 1182. [9] Amer. Jour. Med. Sci., May, 1898. [10] Lancet, April 16, 1898, p. 1042. [11] Practitioner, May, 1898, p. 528. [12] Lancet, June 25, 1898, p. 1747.

